# The Dangers of Misuse of Corticosteroid Drugs in Treating Superficial Fungal Infections: Presentation of a Case Series for Stricter Policy Regulation

**DOI:** 10.1002/puh2.70151

**Published:** 2025-10-24

**Authors:** Fatema Akhter, Sartaz Araf, Mohammad Faisal Hossain, Md. Aminul Haque, Md. Rabiul Islam

**Affiliations:** ^1^ Department of Dermatology and Venereology Bangladesh Institute of Health Science Hospital (BIHS), Mirpur Dhaka Bangladesh; ^2^ Department of Counseling and Education Psychology Texas A&M University Corpus Christi Corpus Christi Texas USA; ^3^ Appalachian College of Pharmacy Oakwood Virginia USA; ^4^ School of Pharmacy BRAC University, Merul Badda Dhaka Bangladesh

**Keywords:** adverse effects, antifungal agents, corticosteroids, drug misuse, fungal infections

## Abstract

Skin fungal infections are a significant global issue and have raised concerns about the effectiveness and appropriateness of using corticosteroids for treatment. This article analyzed 10 case reports of patients in Bangladesh who either received incorrect prescriptions or self‐medicated with different forms of corticosteroids for fungal skin infections. These cases clearly showed the widespread misuse of corticosteroids, resulting in the worsening of fungal infections and posing more health risks. There is an urgent call for improved education for patients, village doctors/medical assistants, and healthcare providers to reduce corticosteroid misuse by promoting proper diagnosis and treatment protocols. Suggestions include using specific, single‐agent topical treatments, enforcing strict regulations, and individualizing prescription durations to optimize patient outcomes and minimize the risks associated with corticosteroid misuse in managing fungal skin infections.

## Graphical abstract

Skin fungal infections are a significant global public health issue. We analyzed 10 case reports of patients in Bangladesh who received different forms of corticosteroids for fungal skin infections. These cases clearly showed the widespread misuse of corticosteroids, resulting in the worsening of fungal infections and posing more health risks. Awareness for patients, village doctors/medical assistants, and healthcare providers to reduce corticosteroid misuse by promoting proper diagnosis and treatment protocols. Using specific, single‐agent topical treatments, enforcing strict regulations, and individualizing prescription durations to optimize patient outcomes and minimize the risks associated with corticosteroid misuse in managing fungal skin infections. Effectiveness and appropriateness of using corticosteroids for treatment of skin fungal infections are crucial.

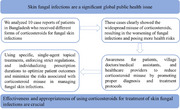

## 1 Background

Superficial fungal infections, such as dermatophytoses and candidiasis, are among the most common skin conditions worldwide, especially in tropical and subtropical regions [1]. Symptoms vary on the basis of the affected area but typically include itching, scaling, and a slightly raised border with minimal inflammation. The rash may be intermittent, with patches that come and go. When inflammation is present, the area may develop fluid‐filled bumps that can ooze [2]. Standard treatment involves topical antifungal creams, with oral antifungal medications reserved for more severe or widespread infections. Although combination antifungal/corticosteroid creams are FDA‐approved for treating tinea corporis, candidiasis, and diaper dermatitis, their use should be approached with caution. Many clinicians are unaware that these products often contain high‐potency corticosteroids, which can lead to skin thinning, delayed healing, and misdiagnosis of steroid‐masked infections if used inappropriately [3].

Topical corticosteroids are highly effective anti‐inflammatory agents, commonly prescribed for conditions such as eczema, psoriasis, and allergic dermatitis [4]. However, when misused in fungal infections, they suppress local immune responses, allowing the infection to spread and leading to intensely inflamed and itchy lesions known as tinea incognito [5]. Such misuse, often due to misdiagnosis or self‐medication, can also mask the typical appearance of fungal infections, making diagnosis more difficult [6]. As a result, treatment may be delayed or misdirected, prolonging the disease course.

The scale of this problem is evident in South Asia. A recent study conducted at a rural tertiary care teaching hospital in Maharashtra, India, found that 140 out of 500 prescriptions contained topical corticosteroids, with 98% of those being very potent formulations. In the majority of cases, there was no clear clinical justification for prescribing the corticosteroids [7]. Easy over‐the‐counter access to potent corticosteroids and combination creams containing both corticosteroids and antifungals has further fueled misuse, particularly in low‐ and middle‐income countries. Many patients rely on advice from informal drug sellers or past experiences, believing these treatments to be more effective because they provide rapid symptom relief [8]. In Bangladesh, poor regulatory control and limited patient counseling have worsened treatment outcomes [9]. Unfortunately, this practice often results in chronic, recurrent, and treatment‐resistant fungal infections [10]. This article presents 10 case reports from Bangladesh showing how inappropriate corticosteroid use—whether due to incorrect prescriptions or self‐medication—can worsen superficial fungal infections.

## 2 Methods

**FIGURE 1:**
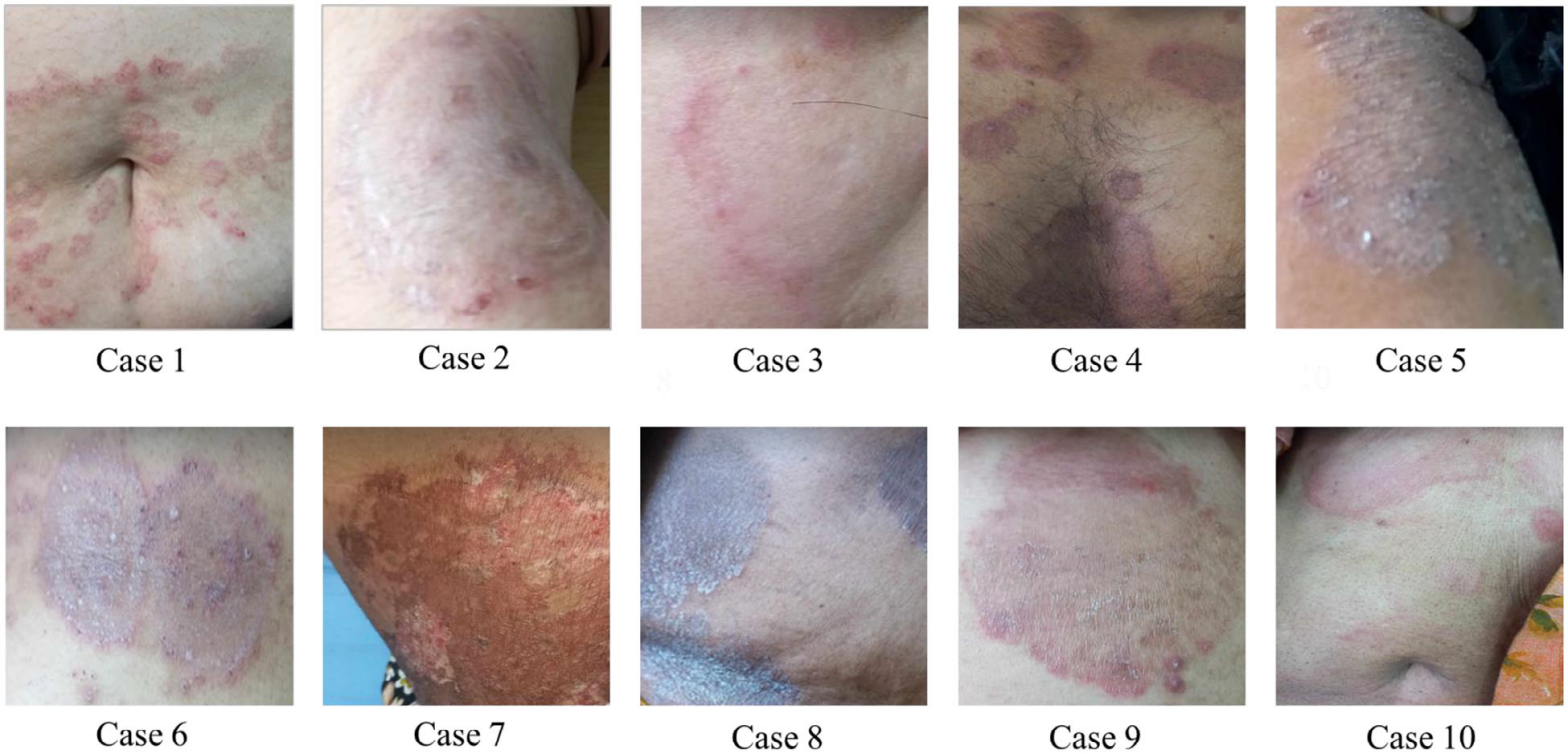
Clinical case presentation to examine the inappropriate use of steroids in the treatment of superficial fungal infections.

The study was conducted at a hospital in the capital city of Bangladesh, focusing on patients diagnosed with superficial fungal infections. Dr. Fatema Akhter, the attending physician and one of the principal investigators, obtained informed consent from the patients for their participation in the study. As part of the investigation, 10 specific cases were identified to examine the inappropriate use of corticosteroids in the treatment of superficial fungal infections. Before starting treatment, Dr. Fatema performed all the necessary blood tests for each case. These cases were thoroughly documented in the case report section, with individual assessments conducted to contribute to the findings of the report (Figure 1).

### 2.1 Case 1

A 50‐year‐old female patient presented with a chronic, widespread fungal infection affecting her groin, axilla, and trunk area for 3 months, characterized by scaling, erythema, and itching. Initially, she attempted homeopathic remedies unsuccessfully. Subsequently, she received five to six doses of intramuscular corticosteroid injections (triamcinolone acetonide) from an unqualified practitioner, causing atrophic skin changes with exacerbation of symptoms. Seeking medical intervention due to the worsening condition, the patient consulted a dermatologist (PI). After proper lab investigation, the dermatologist prescribed the tablet terbinafine 250 mg once daily for 1 month, followed by a weekly capsule of fluconazole 150 mg for 4 weeks, and instructed the application of luliconazole topical cream once daily for 2 weeks. A follow‐up visit was scheduled for 1 month, although the patient returned after 2 months for further assessment. The patient was completely cured.

### 2.2 Case 2

A 31‐year‐old female presented with a widespread fungal infection affecting her abdomen, groin, and both upper and lower limbs. The infection was characterized by scaling and unusual ringworm‐like rashes with double edges and larger skin lesions. The patient initially consulted a dermatologist who prescribed oral corticosteroids for 24 days along with broad‐spectrum antibiotics and multiple antifungal drugs. She was treated with an oral tablet of deflazacort 6 mg for 24 days, a tablet of cefuroxime and clavulanic acid 250 mg twice a day for 21 days, a tablet of itraconazole 100 mg for 2 months, a tablet of terbinafine 250 mg for 2 months, and a tablet of voriconazole 200 mg twice a day for 2 months. However, skin lesions worsened after taking these medications. When her condition continued to worsen despite these treatments, she sought a second opinion and visited the PI, Dr. Fatema. The new prescription included only one antifungal drug, fluconazole 100 mg capsules to be taken once daily for 1 month, along with gentamicin and clotrimazole cream applied twice daily for 1 month. After 1 month of following this new treatment plan, almost all skin lesions had healed. However, the patient returned after 10 days with complaints of itching, although no new skin lesions were observed.

### 2.3 Case 3

A 35‐year‐old diabetic female presented with an atypical pattern of ringworm rashes characterized by erythematous, double‐edged, larger rings on her abdomen, groin, upper and lower limbs, and face. Initially, she had very few lesions, but after taking multiple intramuscular corticosteroid injections and oral corticosteroids, as advised by a salesman at a medicine shop, her condition worsened. Specifically, she had taken weekly injections of triamcinolone acetonide for 9 weeks, deflazacort 24 mg daily for 1 month, terbinafine 250 mg daily for 20 days, itraconazole 100 mg daily for 1 month, and used clobetasol ointment for 2 months. The improper use of corticosteroids led to a significant worsening of her skin lesions and blood sugar levels. Upon visiting Dr. Fatema, she was prescribed fluconazole 100 mg daily and luliconazole topical cream for 1 month. After 1 month, during her follow‐up appointment, she was fully cured with no new lesions.

### 2.4 Case 4

A 43‐year‐old man presented with a widespread tinea infection affecting his neck, chest, axilla, groin, and legs, characterized by redness, scaling, and rounded lesions with intense itching. Initially, his fungal lesions were confined to his groin, axilla, and neck. Following the advice of an unqualified practitioner, he took oral prednisolone 20 mg daily for 3 months and weekly intramuscular injections of triamcinolone acetonide for 6 months, along with fluconazole 50 mg for 20 days. This inappropriate use of corticosteroids led to high blood sugar and exacerbated his skin condition, resulting in larger lesions with increased redness and scaling. Upon visiting Dr. Fatema, she prescribed 100 mg of itraconazole daily in the morning and clotrimazole cream topically for 1 month. At his follow‐up visit after 20 days, he showed marked improvement and was nearly fully recovered.

### 2.5 Case 5

A 27‐year‐old female presented with an extensive and chronic tinea infection affecting her face and nearly all parts of her body, characterized by intense itching, scaling, and distinct ringworm shapes. Despite enduring this condition for 5 years, she had undergone multiple treatments, including oral antifungals, injectable corticosteroids, and oral corticosteroids prescribed by a village doctor. Previous therapies included injectable triamcinolone acetonide weekly for 5 weeks, prednisolone 20 mg orally daily for 1 month, voriconazole 400 mg orally daily for 2 months, and terbinafine 250 mg orally daily for 1 month, all providing only temporary relief before the infection recurred and spread further. Faced with worsening symptoms, she sought a second opinion from Dr. Fatema, who initiated treatment with itraconazole 200 mg orally daily for 1 month along with clotrimazole cream. Within 3 weeks, most lesions had been resolved, except for one on her buttock. Dr. Fatema advised continuing itraconazole for another month to ensure complete eradication of the infection.

### 2.6 Case 6

A 35‐year‐old female presented with a superficial fungal infection on her groin and trunk, characterized by multiple rounded scaling lesions with sharp borders. She had developed these symptoms 2 months prior, initially limited to her groin and waist. She consulted a skin specialist who prescribed 100 mg fluconazole orally for 6 weeks. However, she did not experience significant recovery. Subsequently, she took oral prednisolone 40 mg orally for 15 days and applied clobetasol ointment for 15 days, based on the advice of a salesman. Her condition worsened, with the lesions becoming larger and more widespread, accompanied by intense itching. She then visited Dr. Fatema, who prescribed terbinafine 250 mg orally and luliconazole cream topically for 1 month. Upon follow‐up after 3 weeks, most of the lesions had resolved, although some hyperpigmented patches remained.

### 2.7 Case 7

A 45‐year‐old female presented with an extensive fungal infection covering her body, accompanied by cellulitis on her left arm due to an injectable corticosteroid administered with a faulty technique. She was admitted to the hospital for surgery for cellulitis and subsequently referred to the dermatology department for her skin condition. According to her, the fungal infection initially appeared on her lower abdomen and groin. She had taken multiple medications prescribed by a local physician but could not recall their names. When these treatments failed, she received an intramuscular injection in her deltoid muscle based on the advice of a salesman, which led to a severe infection at the injection site and exacerbated her blood sugar levels, as she is diabetic. Her previous treatment included a triamcinolone acetonide injection. Suffering from the injection site infection, widespread tinea infection, and uncontrolled diabetes, she had to be hospitalized for uncontrolled blood sugar and surgery for cellulitis. After her surgery, she was referred to consult Dr. Fatema for her skin problem. She was prescribed 100 mg of itraconazole orally once daily for 1 month and luliconazole cream twice daily for 1 month. At the first follow‐up after 20 days, the infection was almost cured, and she was advised to continue the same medication. By the second follow‐up, another 20 days later, she was completely cured.

### 2.8 Case 8

A 52‐year‐old female presented with a fungal infection on her waist, abdomen, and axilla, characterized by large, rounded, hyperpigmented scaling lesions with itching for about 6 months. She visited a dermatologist who prescribed two oral antifungals. Additionally, she took an injectable steroid on the advice of a salesman, which may have exacerbated her condition. Her previous prescriptions included terbinafine 250 mg orally twice a day for 1 month, fluconazole orally once a day for 2 months, and two doses of intramuscular triamcinolone acetonide. When she did not experience recovery, she consulted Dr. Fatema and was prescribed itraconazole 200 mg orally once daily for 1 month and clotrimazole cream twice daily for 1 month. Although she did not attend her scheduled follow‐up appointment, she reported over the phone that the infection was completely cured after one and a half months.

### 2.9 Case 9

A 26‐year‐old female patient presented with extensive tinea incognito, characterized by multiple rounded, double‐edged erythematous patches on her abdomen, buttocks, groin, and axilla. She had previously visited a dermatologist who prescribed systemic antibiotics, oral corticosteroids, and oral antifungals. Her medication regimen included deflazacort 24 mg orally once daily for 15 days, cefuroxime + clavulanic acid 500 mg orally twice daily for 15 days, terbinafine 250 mg orally once daily for 15 days, and fluconazole 50 mg orally once daily for 15 days. Despite this treatment, her lesions worsened, prompting her to consult Dr. Fatema. She was prescribed 200 mg of itraconazole orally once daily for 1 month and luliconazole cream once daily for 1 month. During a follow‐up call, 3 months after starting the new treatment, the patient reported that her condition was fully cured.

### 2.10 Case 10

A 48‐year‐old female patient with diabetes mellitus presented with extensive tinea incognito and a secondary infection. Her medication history included deflazacort 6 mg orally twice daily for 1 month, clobetasol ointment for 1 month, and terbinafine 250 mg orally for 2 months, prescribed by a general physician. The use of oral corticosteroids and topical creams exacerbated her lesions. Dr. Fatema, our PI, noted elevated liver enzymes (SGPT: 127 IU/mL) during her initial visit. She prescribed a topical antifungal cream and antihistamine. After 20 days, a follow‐up blood test showed her liver enzymes had normalized to 21 IU/mL. Subsequently, itraconazole 100 mg orally once daily was added for 1 month. At her first follow‐up, there was no significant improvement, so she continued the same medications. By the second follow‐up, her fungal infection had almost been resolved, but she had developed scabies, prompting the addition of a topical permethrin 5% cream for scabies. At her third follow‐up, she still had some residual fungal lesions on her abdomen.

## 3 Discussion

The analysis of 10 case reports from Bangladesh revealed a concerning trend of patients receiving incorrect prescriptions or self‐medicating with different forms of corticosteroids (topical, oral, injectable) for fungal skin infections. These cases highlight the widespread problem of misdiagnosis and inappropriate use of corticosteroids, which worsen fungal infections and pose health risks. The low physician‐to‐patient ratio in developing countries like Bangladesh significantly contributes to these issues. With a shortage of doctors available to serve the population, rushed consultations often lead to inaccurate diagnoses. The World Health Organization recommends at least one doctor for every 1000 patients, but in Bangladesh, each doctor is responsible for an average of 1901 patients, showing the strain on the healthcare system [11, 12].

In public hospital outpatient departments, patients typically wait 1.5 h on average to see a doctor for a consultation lasting under a minute [11, 13]. Recent findings reveal that nearly 80% of individuals frequently turn to drugstores for healthcare services, where unlicensed and unqualified practitioners provide treatment in Bangladesh [11, 14].

Inadequate patient education regarding disease patterns and limited access to knowledgeable community health workers further contribute to the problem. Patients, driven by financial constraints, often resort to self‐medication with corticosteroids, relying on potentially misleading information. Moreover, the involvement of unqualified or underqualified healthcare providers in the form of local community health workers adds to the challenges, as they may lack the necessary expertise to make accurate diagnoses. The cycle of misdiagnosis and misuse continues because patients tend to self‐treat. Effective patient education and counseling are crucial interventions, as shown by their positive impact on patient outcomes in a recent JPCTS study [6].

It is essential to increase patient awareness of the risks of self‐medication and enforce stricter regulations on medication sales, especially for combination products containing corticosteroids. To effectively tackle this issue, we need a comprehensive strategy. This includes improving the physician–patient ratio, providing better education on disease patterns, strengthening the role of community health workers, and improving socioeconomic conditions. Additionally, it is important to enforce medication regulations and promote better guidelines and labeling practices. By making these systemic changes, we can reduce misdiagnosis and misuse of corticosteroids, ultimately leading to better health outcomes for patients.

## 4 Conclusion

The findings from our study highlight the need for thorough education and training on the proper use of topical corticosteroids in combination with antifungal for treating fungal skin infections. It is crucial to educate patients, as well as village doctors, medical assistants, general practitioners (GPs), and dermatologists on the correct application and duration of corticosteroid use. Adhering to these guidelines can help prevent misuse and minimize potential adverse effects. Furthermore, there is a strong need for stricter regulations and enforcement by drug administrations in developing countries to control the prescribing and dispensing of corticosteroids. There should be stricter regulations on the use of injectable and oral corticosteroids. In our study, several patients received these forms of corticosteroids at the advice of village doctors or unlicensed sales personnel. To prevent misuse—particularly in fungal infections—clear prescribing protocols must be established, and medical assistants, village doctors, and general physicians must follow these protocols. Clearer guidelines and monitoring practices can help reduce the inappropriate use of these medications. Additionally, we believe it is best to use individualized topical preparations for antifungal and corticosteroid preparations, instead of combination products. This personalized approach, with specific durations prescribed by physicians, can prevent errors and unnecessarily prolonged treatment, leading to better outcomes for patients. Although these measures may lead to some additional costs, the benefits far outweigh the expenses, especially considering the affordability of corticosteroids and antifungal treatments. By following these recommendations, we can enhance patient safety, improve treatment efficacy, and promote responsible medication practices in the management of fungal skin infections.

## Author Contributions

**Fatema Akhter**: conceptualization, data curation, writing – original draft. **Sartaz Araf**: conceptualization, data curation, writing – original draft. **Mohammad Faisal Hossain**: conceptualization, data curation, writing – original draft. **Md. Aminul Haque**: validation, writing – review and editing. **Md. Rabiul Islam**: conceptualization, supervision, writing – review and editing. The author(s) read and approved the final manuscript.

## Funding

The authors have nothing to report.

## Ethics Statement

All findings of the present cases were consequences of routine clinical evaluation and diagnostics, and further research did not require individual investigations. Moreover, our institution does not require ethical approval for reporting individual cases or case series.

## Consent

Written informed consent was obtained from a legally authorized representative(s) for anonymized patient information to be published in this article.

## Conflicts of Interest

The authors declare no conflicts of interest.

## Transparency Statement

The lead author, Fatema Akhter, affirms that this manuscript is an honest, accurate, and transparent account of the study being reported; that no important aspects of the study have been omitted; and that any discrepancies from the study as planned (and, if relevant, registered) have been explained.

## Data Availability

The data that support the findings of this study are available from the corresponding author upon reasonable request.
